# Dr. Ernest Wethered

**Published:** 1910-05-07

**Authors:** 


At the early age of 34 Dr. Ernest Wethered has died at Kingswood, Surrey. He took his diploma in 1899, his M.B. with honours in 1902, and became an M.D. of London in 1904. He had been a student at Bart's, and was at one time house surgeon to the Royal Infirmary, Derby. His other appointments included those as various as civil surgeon to the South African Field Force and house physician at St. Bartholomew's Hospital.

